# Loss of the Y Chromosome: A Review of Molecular Mechanisms, Age Inference, and Implications for Men’s Health

**DOI:** 10.3390/ijms25084230

**Published:** 2024-04-11

**Authors:** Itzae Adonai Gutiérrez-Hurtado, Astrid Desireé Sánchez-Méndez, Denisse Stephania Becerra-Loaiza, Héctor Rangel-Villalobos, Norma Torres-Carrillo, Martha Patricia Gallegos-Arreola, José Alonso Aguilar-Velázquez

**Affiliations:** 1 Departamento de Biología Molecular y Genómica, Centro Universitario de Ciencias de la Salud, Guadalajara 44340, Jalisco, Mexico; 2 Laboratorio de Ciencias Morfológico Forenses y Medicina Molecular, Departamento de Morfología, Centro Universitario de Ciencias de la Salud, Guadalajara 44340, Jalisco, Mexico; 3 Doctorado en Genética Humana, Departamento de Biología Molecular y Genómica, Centro Universitario de Ciencias de la Salud, Universidad de Guadalajara, Guadalajara 44340, Jalisco, Mexico; 4 Departamento de Aparatos y Sistemas II, Universidad Autónoma de Guadalajara, Zapopan 45129, Jalisco, Mexico; 5 Instituto de Investigación en Genética Molecular, Departamento de Ciencias Médicas y de la Vida, Centro Universitario de la Ciénega, Universidad de Guadalajara, Ocotlán 47820, Jalisco, Mexico; 6 Departamento de Microbiología y Patología, Centro Universitario de Ciencias de la Salud, Universidad de Guadalajara, Guadalajara 44340, Jalisco, Mexico; 7 División de Genética, Centro de Investigación Biomédica de Occidente (CIBO), Instituto Mexicano del Seguro Social (IMSS), Guadalajara 44340, Jalisco, Mexico

**Keywords:** mosaic loss of Y chromosome, men’s health, Alzheimer’s disease

## Abstract

Until a few years ago, it was believed that the gradual mosaic loss of the Y chromosome (mLOY) was a normal age-related process. However, it is now known that mLOY is associated with a wide variety of pathologies in men, such as cardiovascular diseases, neurodegenerative disorders, and many types of cancer. Nevertheless, the mechanisms that generate mLOY in men have not been studied so far. This task is of great importance because it will allow focusing on possible methods of prophylaxis or therapy for diseases associated with mLOY. On the other hand, it would allow better understanding of mLOY as a possible marker for inferring the age of male samples in cases of human identification. Due to the above, in this work, a comprehensive review of the literature was conducted, presenting the most relevant information on the possible molecular mechanisms by which mLOY is generated, as well as its implications for men’s health and its possible use as a marker to infer age.

## 1. Introduction

It is known that the Y chromosome determines sex in men, and that throughout the evolution of our species it has been degenerating. It has lost approximately 1500 genes and only conserved 106 genes that code for certain proteins related to the development of the testes and spermatogenesis, as well as several pseudogenes and noncoding RNAs [1,2,3,4]. Among the well-known Y chromosomal genes are included SRY and SHOX, which are involved in testicular development and skeletal growth, respectively. In addition, multicopy genes at Yq11 mediate spermatogenesis. In contrast, Y chromosomal genes play no significant role in the survival or mitosis of somatic cells [1]. Hence, a human male somatic cell can survive and proliferate even when it loses the Y chromosome. Consequently, 45, X cells gradually accumulate in the bodies of men.

**Figure 1:**
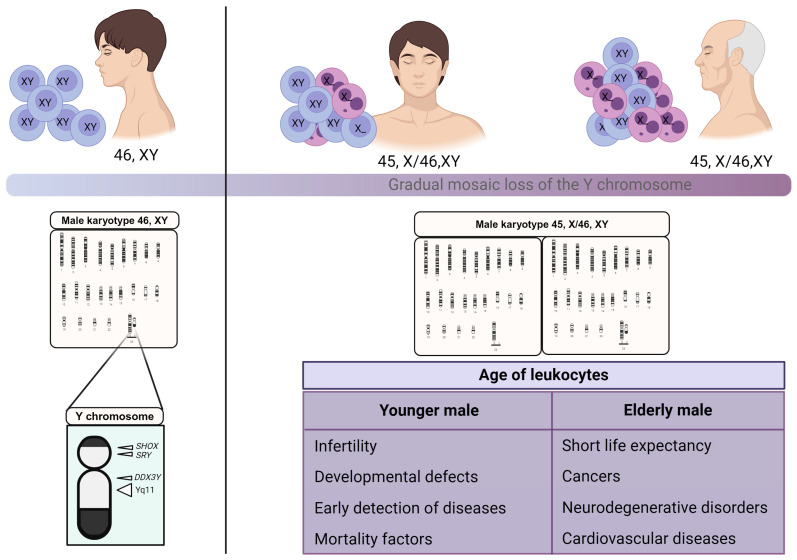
Implications of mLOY in men. Normally, men’s karyotype configuration is 46, XY (**left**); however, throughout life, subsets of men’s cells gradually lose the Y chromosome (karyotype: 45, X/46, XY), which is associated with different pathological conditions, both in younger and elderly males (**right**).

Recent studies have shown that mosaic loss of the Y chromosome (mLOY) is a common feature in the blood of elderly men [5,6]; however, it is associated with short life expectancy, cancers, and other disorders [1,2,3,4,5,6,7]. On the other hand, mLOY in men at younger ages, although rare, is likely to underlie infertility and developmental defects [8] (Figure 1). In recent years, multiple epidemiological studies have shown that mLOY in somatic cell subsets at different stages of postzygotic development and throughout life is associated with mortality factors in males and serves as a marker for early detection of disease [9]. Genes of great relevance to these aspects have been observed, such as DDX3Y, which modulates cell differentiation development [10], and long non-coding RNAs promote the development of prostate cancer and play a role in processes important for the development of fatty liver, cellular inflammation due to atherosclerosis, and cardiovascular diseases [4].

mLOY is the most common chromosomal change observed in leukocytes in elderly men [11,12]. For a long time, mLOY was considered a physiological phenomenon associated with aging. This theory was mainly based on the idea that none of the known genes on the Y chromosome are essential for cellular viability, as well as the observation that women can survive without this chromosome [1]. However, it has now been proposed that mLOY may be related to male infertility, as well as neurodegenerative disorders such as Alzheimer’s disease, cardiovascular diseases, autoimmune diseases, increased risk of overall mortality, and especially with cancer [5,13,14,15]. However, there have been no literature reviews that analyze in depth the molecular mechanisms that produce mLOY. Therefore, this paper aims to review the molecular mechanisms that may be associated with Y chromosome loss in males, as well as the effects of mLOY on men’s health and its possible application in forensic genetics (Figure 1).

## 2. Molecular Mechanisms of the Loss of the Y Chromosome

The precise causes of mLOY are still not fully understood. However, there are several proposed mechanisms. One of the hypotheses with significant scientific support suggests that mLOY may result from errors in chromosomal segregation during mitosis; such errors are known to cause aneuploidy [16]. It has been suggested that the unique structure of the Y chromosome, which includes palindromic sequences that may facilitate the formation of isodicentric chromosomes, as well as its stability and propensity for centromere dysfunction, could make it more susceptible to segregation errors [1,17].

**Figure 2:**
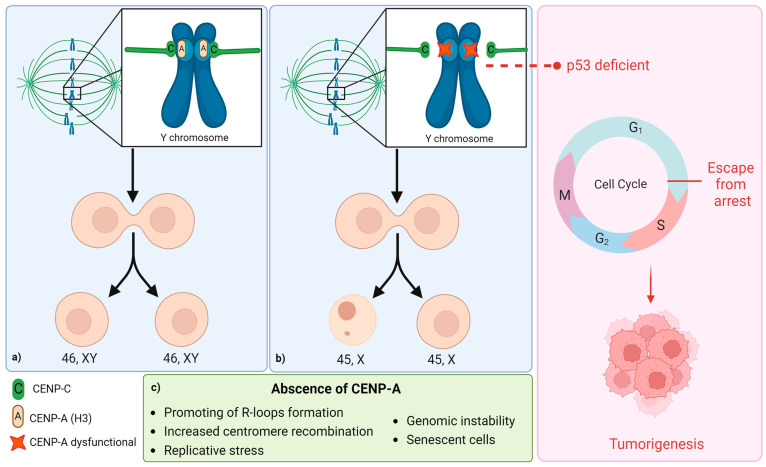
Mechanisms of absence or failure of CENP-A associated with mLOY. During cell division, in the Y chromosome, the kinetochores assemble to centromeric chromatin by CENP-C, which binds to CENP-A (**a**). When this binding fails, the Y chromosome is more prone to segregation defects, causing mLOY (**b**). Thus, the absence or failure of CENP-A may trigger mLOY through different mechanisms (**c**). Interestingly, the protein CENP-B, which is not present in the Y chromosome, enhances the recruitment of CENP-A to reinforce the binding of CENP-C to the centromere and thus make the mitotic process more efficient. Therefore, dysfunction of CENP-A alone could affect the kinetochore binding to the centromere of the Y chromosome, making this chromosome more prone to chromosomal segregation defects. On the other hand, the absence of CENP-A in p53-deficient cells can lead to tumorigenesis (**right**).

During mitosis, kinetochores assemble on the outer surface of centromeric chromatin through nucleation by the centromere protein C (CENP-C), which binds to the centromere via the histone H3 variant known as centromere protein A (CENP-A). This CENP-A binding occurs specifically at the suprachromosomal family 1 (SF1), which is a tandem array of two alternate units of α satellites. In addition to CENP-A, another protein of great importance in this process is the centromere protein B (CENP-B), which also binds to SF1 dimers. It has been proposed that CENP-B binding enhances the recruitment of CENP-A, acting as a “backup” mechanism to reinforce the binding of CENP-C to the centromere and thus make the mitotic process more efficient [16,18,19]. However, it is known that CENP-B is present on all chromosomes except the Y chromosome. Therefore, dysfunction of CENP-A alone could affect the kinetochore binding to the centromere of the Y chromosome, making this chromosome more prone to chromosomal segregation defects [16,19,20,21] (Figure 2).

In addition to its role in binding to the kinetochore, CENP-A removal during the S phase of the cell cycle has been observed to significantly increase centromere recombination. It is hypothesized that the absence of CENP-A may promote the formation of R-loops, possibly due to the convergence of replication and transcription mechanisms, consequently leading to increased centromere recombination. This heightened recombination at the centromere could induce replicative stress, a well-known trigger of genomic instability. Understanding this instability could provide valuable insights into the relationship between mLOY and diseases such as cancer, making it a promising area for future research [22].

Given the unique characteristics of the Y-chromosome centromere, investigating its integrity is crucial for future research on mLOY. A comprehensive understanding of CENP-A function is essential in this context to grasp centromere stability and functionality. It has been confirmed that mitotic centromeres typically contain between 300 and 400 CENP-A molecules [18,23]. Additionally, a decrease in CENP-A levels has been observed in senescent cells, and this reduction in fibroblasts leads to premature senescence. Notably, mLOY is more common in elderly individuals, mirroring the decrease in CENP-A levels with aging [1]. These phenomena may be concurrent and interdependent; however, to date, the levels or functionality of CENP-A and mLOY have not been studied simultaneously.

Another crucial factor for future research is the role of p53 functionality in individuals with mLOY. The presence of CENP-A only in cells with deficient p53 function leads to improper chromosomal segregation. Functional p53, on the other hand, halts mitosis in defective cells. Improper chromosomal segregation due to CENP-A depletion, coupled with p53 deficiency, may be a significant mechanism in the tumorigenesis associated with aging [1,24] (Figure 2).

Currently, it is established that the Y chromosome contains 693 genes and 883 transcripts, with 106 predicted to encode proteins [2]. However, the definitive role of any Y-chromosome genes in diseases associated with mosaic loss of the Y chromosome (mLOY) remains unclear. This uncertainty highlights the importance of future research to elucidate this aspect. An essential focus for future investigations is to determine whether mLOY acts as a risk factor for certain diseases or merely occurs concurrently with them.

From our perspective, it is plausible that mLOY is closely linked to cellular senescence. This hypothesis arises from the shared risk factors between LOY and cellular senescence, such as exposure to air pollution, smoking, obesity, and alcohol consumption. These factors can induce cellular changes contributing to both cellular senescence and LOY [1,17,25,26]. Thus, there exists a potential association between mLOY and cellular senescence that warrants further investigation to comprehensively understand their relationship and implications for human health.

## 3. Possible Fate of Y Chromosome after It Is Loss

**Figure 3:**
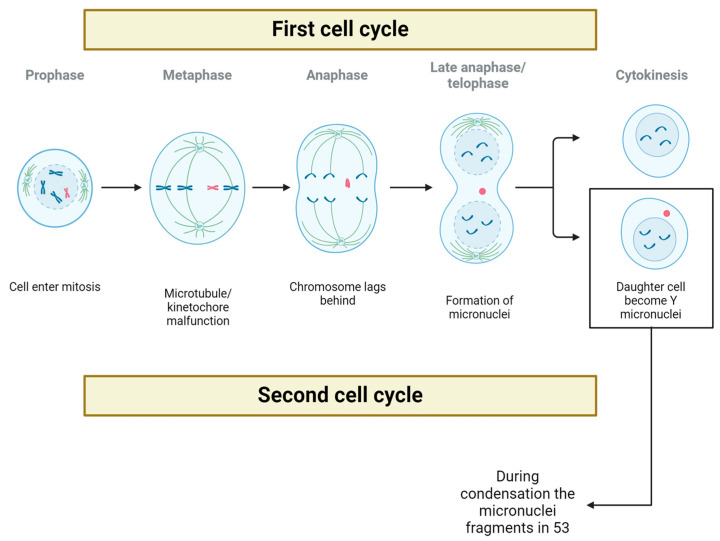
Effects of mLOY on cells. In CENP-A deficient cell models, it has been observed that a micronucleus is formed that houses the unsegregated Y chromosome, and by the second cycle of mitosis, this micronucleus is fragmented into 53 parts.

In vitro models utilizing dysfunctional CENP-A revealed errors in Y-chromosome segregation. This led to the formation of a micronucleus derived from the Y chromosome during the first cell cycle. Subsequently, in the second cell cycle, the Y chromosome was observed to fragment into 53 distinct fragments, probably resulting from the condensation of the initial micronucleus [16] (Figure 3).

Numerous studies have demonstrated that an increased frequency of micronuclei is associated with a higher risk of cancer and various age-related diseases [27,28]. Similarly, mLOY has been linked to aging and a predisposition to diseases [14]. Given these findings, it is plausible to suggest that both the presence of micronuclei and mLOY could serve as markers of genomic stress and be associated with aging processes and related pathologies.

## 4. Other Factors Associated with the Loss of the Y Chromosome

The longitudinal progression of mLOY varies among elderly men [29]. Thus, mLOY is likely to be enhanced or suppressed by various environmental and genetic factors. Of these, tobacco smoking represents the major risk factor for aging-related mLOY [6]. Since the frequency of mLOY in current smokers is significantly higher than that in former smokers [30,31], the effect of tobacco smoking on mLOY appears to be reversible. In addition, several other environmental factors, such as polycyclic aromatic hydrocarbons, air pollution, heavy drinking, and obesity, have been linked to the risk of mLOY [1]. Yet, the significance of these factors needs to be confirmed in future studies. Multiple SNPs in the genome have been associated with the risk of aging-related mLOY [11,32]. These SNPs include variants in genes involved in cell cycle regulation, tumor growth, and cancer susceptibility, and may facilitate Y-chromosomal loss during mitosis or clonal expansion of 45, X cell lineages. In addition, structural alterations of the Y chromosome have also been linked to the risk of mLOY. In particular, large deletions on the Y chromosome are frequently coupled with 45, X/46, XY mosaicism [33,34]. Interestingly, another study indicated that common copy-number variations in the azoospermia region at Yq11 did not increase the frequency of mLOY [35].

## 5. Techniques for Detection of Loss of the Y Chromosome

Various molecular techniques can be utilized to detect mLOY. Initially, conventional cytogenetic techniques can be employed to obtain the karyotype of the individual, followed by staining the chromosomes with G bands. Subsequently, fluorescence in situ hybridization (FISH) can be used to identify total or partial chromosomal losses in the cells. This method involves employing a control probe and a probe targeting a gene of interest, such as SRY, to compare their presence or absence. This approach has confirmed a significant incidence of Y-chromosome loss in peripheral blood cell samples.

In addition, other studies have employed more advanced methods, such as next-generation sequencing (NGS), including SNP array or quantitative PCR (qPCR) [29]. Multiplex PCR, utilizing specific markers for the Y-chromosome loci of interest, has also been utilized. Droplet digital PCR (ddPCR) complements these analyses and the results obtained are refined by implementing multiplex ligation-dependent probe amplification (MLPA). This comprehensive approach allows the evaluation and comparison of various loci on the Y chromosome. Importantly, these methodologies enable the accurate detection of chromosomal variations in large cohorts, providing a valuable tool for population-scale research [17].

## 6. Implications of Loss of the Y Chromosome for Men’s Health

In somatic cells, mLOY has emerged as a genetic marker that has been linked to a variety of health conditions, including cancer, cardiovascular diseases, and Alzheimer’s [1]. Although the association of mLOY with these diseases has been studied, research suggests that its implication goes beyond this, encompassing a broader spectrum of pathological conditions. Currently, it is necessary to further explore the role of mLOY in various diseases and its potential as a predictive biomarker and the development of specific therapies in the presence of mLOY. Below are some of the conditions that have been associated with mLOY.

### 6.1. Loss of the Y Chromosome and Alzheimer Disease

In the central nervous system, mLOY is a phenomenon with vast areas to explore. In 2001, Rehen and colleagues were pioneers in revealing the presence of chromosomal aneuploidy in both developing and adult neurons [36]. Aneuploidy in the central nervous system is a remarkably intriguing phenomenon. Unlike most cases of aneuploidy, which are usually associated with processes of mitotic cell division, the brain predominantly harbors cells that are not actively dividing [37].

**Figure 4:**
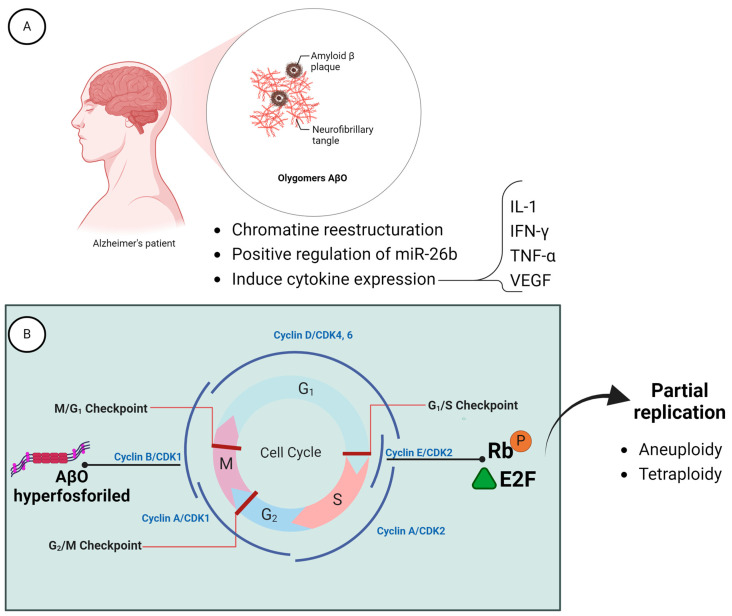
The effect of AβO on the cell cycle of hippocampal neurons. It can be observed that AβOs, by inducing chromatin restructuring, positive regulation of miR-26b, and expression of IL-1, IFN-γ, TNFα, and VEGF, modify the activity of the Cdk2-cyclin E complex (**A**), allowing progression from the G1 to the S phase through Rb phosphorylation and, consequently, activation of E2F. Although partial or total replication occurs, generating tetraploid or aneuploid cells, mitosis does not take place (**B**). This is because the Cdk1/cyclin B1 complex cannot enter the nucleus to trigger envelope-breaking events, as it remains in the cytoplasm associated with hyperphosphorylated tau oligomers.

A particular situation is Alzheimer’s disease (AD), where affected neurons have been observed to undergo DNA replication [38,39]. The regulation of replication in neuronal cells could be due to the toxic oligomers of β-amyloid (AβO), which induce an increase in IL-1, IFN-γ, TNFα, and VEGF, possibly stimulating neurons to attempt to restart the cycle in the hippocampus. It has been proposed that AβO, through chromatin restructuring and positive regulation of miR-26b, modifies the activity of the Cdk2-cyclin E complex, which in turn affects the neuronal regulation of E2F and Rb (retinoblastoma), thus allowing progression from the G1 to the S phase. Although these neurons may re-enter the cell cycle, mitosis is rarely observed in them, due to the interaction of hyperphosphorylated tau oligomers with the Cdk1/cyclin B1 complex in the cytoplasm, which prevents this complex from entering the nucleus to initiate mitosis. As a result, cells become trapped in the G2/M transition phase (Figure 4), leading to a tetraploid condition or aneuploidies. Despite this anomalous state, these cells can survive for long periods in the affected brain [40,41,42].

In patients with AD, it has also been observed that the frequency of mLOY is significantly higher in micro glial cells of patients compared with cells from healthy individuals. In this regard, the frequency of mLOY was found to be 28.1% in AD patients, whereas it was only 2.51% in healthy controls [43,44]. On the other hand, mLOY is considered a rare event in neurons, astrocytes, and oligodendrocytes [44]. It has been observed that, unlike neurons where the Cdk1 enzyme cannot initiate mitosis, in microglia, lesions caused by the disease induce a temporally positive regulation of Myc. This regulation, in turn, stimulates a positive activation of Cdk1, facilitating an initial phase of microglial proliferation in response to the injury [41,45].

It is likely that the mechanisms leading to polyploidy in neurons of AD patients are like those promoting proliferation and LOY in microglial cells. This topic deserves thorough investigation in future studies. Although the precise relationship between AD and LOY has not yet been established, the association appears to be consistent. Since the publication in 2016 of the first study that found an association between LOY and AD, up to the present date, six articles consistently supporting this association have been published, with these studies mainly utilizing peripheral blood as the unit of analysis [29,44,46,47,48,49].

### 6.2. Loss of the Y Chromosome in Kidney Disease

The relationship between mLOY and renal function has been known for years. Initially, mLOY was thought to be a marker for certain types of renal tumors, such as renal papillary cell adenomas or some types of cancer. However, it was soon discovered that mLOY can be found in renal tissue of end-stage renal diseases and not exclusively in neoplasms [50,51].

The presence of LOY varies depending on the location in the nephron, with proximal tubule cells being the most affected. This cell type, predominant in the renal cortex, is highly vulnerable to hypoxic injury, triggering cell de-differentiation and division for epithelial regeneration. Because of constant injury and repair events in these cells, DNA damage may occur, promoting the development of mLOY [12]. This phenomenon coincides with observations indicating that papillary renal cell carcinoma, a renal tubular epithelial tumor, frequently exhibits mLOY compared with other types of renal cancer [52,53]. Although the exact mechanism that generates LOY is currently unknown, it is possible to speculate that, due to the unique centromere structure of the Y chromosome, which makes it more susceptible to chromosomal missegregation during mitosis, the more a cell divides, the more likely it is to experience LOY. As a result, highly proliferative cell types that are more exposed to differentiation and division processes may be particularly susceptible to LOY [12].

Although renal cancer is more prevalent in men than in women, this difference, rather than being due to mLOY, may be influenced by risk factors such as smoking and hypertension, which are more common in men [54]. It has been suggested that, although women may experience DNA damage, they may not exhibit such a clear signal as mLOY. One possible explanation could be that loss of the X chromosome (LOX), although it is associated with female aging, occurs much less frequently than LOY. Therefore, it is proposed that the presence of LOY could be more an indicator of DNA damage and cellular senescence than a risk factor for kidney disease [12].

### 6.3. Loss of the Y Chromosome in Cardiovascular Disease

The association between mLOY and cardiovascular diseases has been widely documented [55,56,57,58]. In the specific case of cardiovascular diseases, studies in animal models and in humans have shown that mLOY is linked to an increase in mortality from heart failure. Even in individuals undergoing transcatheter aortic valve replacement, an increase in cardiovascular risk associated with mLOY has been observed [56,57,59].

**Figure 5:**
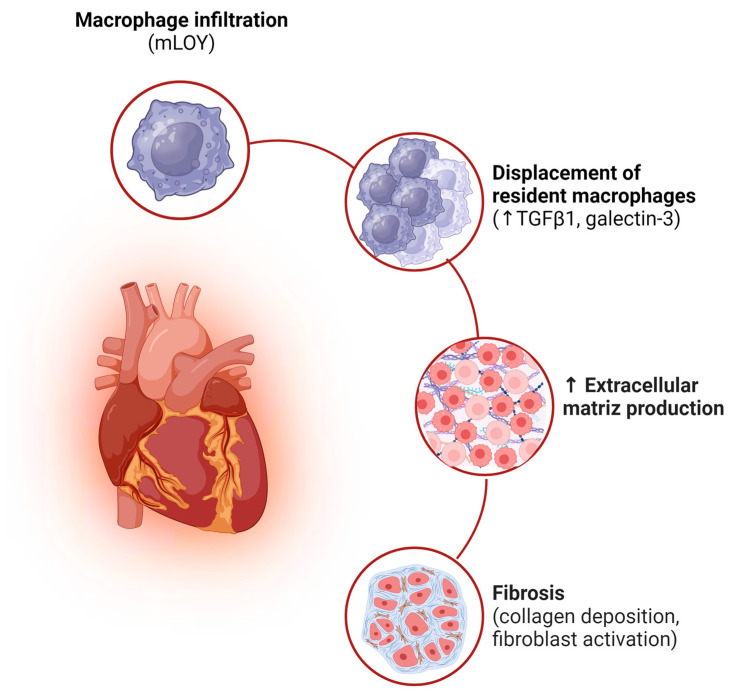
Implications of mLOY in heart function. As a result of a cardiac injury or the aging process, macrophages carrying LOY can infiltrate the heart and replace resident macrophages. These LOY-deficient macrophages exhibit overexpression of TGFβ1 and galectin-3, which contributes to the excessive proliferation and activation of cardiac fibroblasts, as well as the increased production of extracellular matrix. This process negatively affects cardiac function.

Regarding the pathophysiological mechanism that links mLOY to cardiovascular disease, its possible involvement in the development of fibrosis has been suggested [55]. This phenomenon may result from cardiac macrophages, originating from Y chromosome-deficient hematopoietic stem cells, presenting alterations in their functions. The affected macrophages can infiltrate the heart in response to different types of cardiac injuries or replace yolk sac-derived macrophages residing in the heart during aging. Macrophages carrying LOY show excessive activation of a signaling network that promotes fibrosis. This activation is characterized by the overexpression of regulons associated with transforming growth factor-β1 (TGFβ1) signaling, as well as an increase in the expression of galectin-3, known for its profibrotic effects. Because of LOY deficiency in macrophages, there is excessive proliferation and activation of cardiac fibroblasts, as well as excessive production of extracellular matrix, leading to a decrease in cardiac function [57] (Figure 5).

### 6.4. Loss of Y Chromosome and Cancer

The relationship between the Y chromosome and cancer is a complex field of research that deserves further investigation. It is well known that, in most cases, men have a higher incidence of cancer compared with women at shared anatomical sites. Although initially this difference could be attributed to behavioral risk factors such as smoking or alcohol consumption, disparities in the frequency and severity of cancer between both sexes persist even when considering these factors in analyses. This suggests that other biological sex-related factors may be playing a crucial role in this discrepancy [60].

Currently, an association has been established between LOY (loss of Y chromosome) and various types of cancer, such as bladder cancer, prostate cancer, mesothelioma, glioblastoma, and colorectal cancer. Surprisingly, this chromosomal loss has also been observed to be related to breast cancer in men. The underlying molecular bases explaining how LOY may increase the risk and severity of cancer are diverse and are still being investigated in depth [13,61,62,63,64,65,66].

Bladder cancer is one of the types of cancer that has been the subject of extensive research on the possible molecular pathways through which LOY could increase cancer risk. It has been observed in studies with animal models and in vitro models that LOY is associated with the creation of an immunosuppressive tumor microenvironment. This microenvironment is characterized by the depletion of CD8 T cells [67]. This finding is congruent with previous research, which had also indicated that patients with advanced-stage bladder cancer exhibited a decrease in the percentage of CD8 T lymphocytes [68]. The association between LOY and the decrease in CD8 T cells could also be a relevant indicator for treatment selection. Since the depletion of CD8 T cells correlates with a more favorable response to anti-PD-1 immunotherapy, the presence of LOY, being associated with the depletion of CD8 T cells, could influence therapeutic decision-making [66,67].

Given the established relationship between LOY and CD8 T cells, it would be interesting for future research to also explore the association between LOY and CD39. CD39 is an ectoenzyme expressed by CD8 T cells that plays a crucial role in regulating the tumor microenvironment. This enzyme is responsible for converting ATP into adenosine diphosphate (ADP) and cyclic adenosine monophosphate (cAMP), ultimately leading to the generation of extracellular adenosine. This adenosine has immunosuppressive properties and can significantly contribute to the inhibition of the anti-tumor immune response in the tumor microenvironment [69].

Other factors that could contribute to the relationship between loss of the Y chromosome (LOY) and cancer are certain proteins encoded by specific genes on the Y chromosome. Among these proteins, CD99 stands out, as well as the histone demethylase KDM5D [66,70,71]. CD99 is an immunoprotein encoded in the pseudoautosomal region 1 of the Y chromosome, expressed on the cell surface of leukocytes. This protein plays various key functions in processes such as transendothelial migration, cell adhesion, differentiation, apoptosis, and intracellular trafficking of proteins related to immune surveillance. The decrease in CD99 is believed to alter the function of immune system cells, potentially favoring cancer development [72].

KDM5D, also known as JARID1D, is a histone demethylase associated with the suppression of the invasive capacity of prostate cancer cells. It has been observed that KDM5D negatively regulates the expression of multiple matrix metalloproteinases (MMPs), including MMP1, MMP2, MMP3, MMP7, MMP10, MMP13, and MMP14, which are related to tumor cell invasion [71]. In comparison with normal prostate tissues, primary prostate tumors have been found to have low levels of KDM5D, while metastatic prostate tumors exhibit even lower concentrations than primary tumors. This pattern suggests that the decrease in KDM5D could contribute to the development and progression of prostate cancer, facilitating invasion and metastasis [71].

Studies on the impact of LOY on cancer, including its development, progression, and treatment, are in an early stage. Although there is increasing evidence suggesting a close relationship between LOY and cancer, much remains to be explored. Most of the evidence points to immune system-mediated mechanisms possibly explaining this relationship, as mLOY appears to make cancer cells less susceptible to immune system attacks.

## 7. Loss of Y Chromosome as a Potential Marker to Infer Men’s Age

Routinely, forensic genetics employs short tandem repeats (STRs) to solve human identification (HID) casework. However, these markers do not always allow human identification, especially when the DNA’s integrity is compromised or when the genetic profile information is insufficient to solve the case. In these situations, forensic geneticists must make use of other tools, like ancestry-informative or phenotype-inference markers. When genetic markers (mainly SNVs) are employed to infer externally visible characteristics, it is known as forensic DNA phenotyping (FDP) [73].

There is a broad spectrum of features that have been analyzed for potential use as part of FDP, including the following: (1) inference of iris, hair, and skin color by using SNV markers in key genes [74,75,76]; (2) eyebrow color [77]; (3) freckle presence [78]; (4) hair shape [79]; (5) hair loss in males [80]; and (6) body height [81]. Even the inference of other habits has been reported; for example, the inference of tobacco and alcohol consumption habits based on DNA methylation (DNAm) analysis from blood [82] or the genetic identification of the genital microbiome exchanged during sexual acts (named “sexome” by the authors), which may be useful in sexual assault cases [83].

Another aim of great interest in the forensic area is to infer the age of a person through the DNA from a biological sample, which can be very useful for the purpose of discrimination among potential donors of the sample. Moreover, age inference is of great interest in the forensic field because the correct inference of other traits (hair color, body height, hair loss, among others) depends directly on the sample donor’s age [84]. Currently, several studies have been published addressing age inference based on a variety of methodologies and genetic sources [85,86,87,88,89,90,91,92,93,94,95,96,97,98,99,100,101,102,103,104,105,106,107,108,109,110,111,112,113,114,115,116,117,118,119,120,121,122,123,124,125,126,127,128,129,130,131,132]. Interestingly, only DNAm analysis has proved to be sufficiently accurate to provide a practical solution for forensic applications [85,86,87,88,89,90,91,92,93,94,95,96,97,98,99,100,101,102,103,104,105,106,107,108,109,110,111,112,113,114,115,116,117,118,119,120,121,122,123,124,125,126,127,128,129,130,131,132], although it requires bisulfite conversion, which is time-consuming and needs high DNA input.

Among the other systems that have not been thoroughly studied as possible markers for inferring age from a DNA sample are telomere shortening and mLOY. Telomere shortening has great potential [133], since the laboratory techniques are easy to standardize. Still, the accuracy reported is low, equivalent to the accuracy of using classical anthropological methods to infer age [134]. On the other hand, mLOY has peculiar characteristics that could enhance its use in this area. For example, it is the most common non-physiological postzygotic genetic alteration in human beings [7,135] and is a normal aging process, since it is present in healthy individuals and its percentage rises over time [136]. Moreover, a mean decrease of 5.5 years in the lifespan of men who presented mLOY in their blood cells was previously observed [5]. Therefore, mLOY can be seen as a biological age marker, and its presence has the potential to be used as a predictive biomarker of male age-related diseases [47].

The analysis of the Y chromosome is commonly performed in forensic casework, and the application of mLOY analysis can provide both disadvantages and advantages in this field. On the one hand, mLOY may interfere with the forensic analysis of male samples because the detection of the Y chromosome could be compromised. On the other hand, mLOY can be of use in forensic cases by adding information that can be brought to the law court as well.

Biological age reflects the aging state that evaluates health status, and a quantification based in numbers cannot be given [137]. Biological age is more a predictor of lifespan than a predictor of chronological age (time that has passed since birth) [138]. The frequency of LOY is age-related; males under 50 years old have low mLOY frequencies contrasting with the fast increase in the percentage of mLOY by the age of 80 and beyond [31].

In a recent study, research was conducted on the ability to predict age using the percentage of mLOY through ddPCR, where 232 male samples (blood, saliva, and semen) from healthy volunteers of different ages were analyzed. When considering age as an integer, the correlation was not stable. However, when calculating the average percentage of mLOY in each age group, a stronger correlation was observed, suggesting that, while not a precise marker at the individual level, mLOY could provide useful information for predicting age based on age groups. Contrastingly, in saliva and semen samples, no significant correlations were found between the percentage of mLOY and age, indicating that this marker may not be effective for predicting age in these samples. Therefore, while there is a significant correlation between mLOY and age in blood samples when considering age groups, this correlation is not evident in saliva and semen samples. Together, these results limit the utility of mLOY as a biomarker of aging specifically to certain types of biological samples and provide relatively good performance in age predictions based on age groups [139]. However, these results must be interpreted with caution due to the following reasons: (A) a relatively small sample size (n = 232); (B) the samples’ unique ethnic origin, as they were all from the Chinese Han group; (C) the analysis of samples using insertion/deletion markers of AMELY and AMELX genes via ddPCR. Although the study’s methodology is adequate, it has the limitation of potential drop-out of insertion/deletion alleles due to mutations in the primers’ annealing sites (silent alleles), which could lead to an underestimation of mLOY. Additionally, (D) samples from various sources (blood, saliva, and semen) were collected from different individuals. Investigating biological samples of diverse types (blood, saliva, semen, etc.) from the same individual can offer further insights into the characteristics of mLOY and may elucidate its underlying mechanism [139].

Before considering the use of mLOY as a marker for inferring the age of individuals in forensic cases, several limitations need a thorough investigation. Some of these limitations include the following: (1) mLOY is exclusively detected in men, therefore age inference from samples of women should be disregarded; (2) although mLOY generally increases with age, there is no consistent pattern and it varies significantly among individuals [29], complicating age inference; (3) in forensic cases where mLOY is present, a significant reduction in genetic markers linked to the Y chromosome (Y-STRs and Y-SNPs) is expected, potentially leading to errors in detection of these markers; (4) many forensic cases, especially those involving sexual abuse, rely on semen analysis to identify male perpetrators. However, the absence of mLOY in spermatozoa has been reported [139]. An alternative approach could be searching for mLOY in Y chromosomes of male epithelial cells in the victim’s body. Nonetheless, the limited quantity of these cells typically found in such cases presents a challenge in mLOY detection.

To address the above mentioned issues, it is imperative to undertake comprehensive studies involving substantial numbers of biological samples (such as blood and saliva samples) from the same individuals across diverse age groups and ethnic backgrounds globally. This approach could aim to develop a more precise algorithm and evaluate the efficacy of mLOY as an age inference marker. Alongside other methods like DNA methylation, this tool holds promise for enhancing human identification in forensic investigations from a comprehensive perspective.

## 8. Conclusions

The gradual accumulation of mLOY is a feature that appears to be age-related in the cells of older men. Various studies have demonstrated the association of mLOY with different pathologies in men, such as Alzheimer’s disease, kidney disease, cardiovascular diseases, and various types of cancer. However, the exact molecular mechanisms underlying mLOY remain unknown. It has been suggested that the interaction between CENP-C and CENP-A proteins may be responsible for mLOY, but further studies are needed to elucidate this mechanism and its relationship with p53 dysfunction in age-associated tumorigenesis cases.

On the other hand, the study of mLOY for inferring the age of males has emerged as a topic of great interest in forensic sciences and human identification. However, several limitations require thorough investigation before the application of mLOY in determining the age of individuals in forensic cases can be considered reliable. In summary, mLOY appears to play a role in a wide range of pathological conditions and could potentially aid in age inference for individual identification, but additional research is essential to advance our understanding and contribute solid data to the existing knowledge.
